# Public Health.—Mr. Buckingham's Bill for Establishing Public Walks, Play-Grounds, Baths, &c.

**Published:** 1836-01

**Authors:** 


### PUBLIC HEALTH.

### *Mr. Buckingham's Bill for establishing Public Walks, Baths, &c.*

A Bill was brought into Parliament by Mr. Buckingham, during the last session, to facilitate the formation and establishment of public walks, playgrounds, baths, and places of healthy recreation and amusement in the open air, in the neighbourhood of such cities, boroughs, and towns, as may require them for the use and accommodation of their inhabitants. The design of this Bill is excellent. With the exception of good foot-paths along the most frequented public roads in the neighbourhood of great towns, there is the most perfect disregard shown towards all that part of the population which is condemned to take exercise on foot. Parks and pleasure-grounds are rigorously locked up, commons are enclosed, foot-paths are interdicted, and the mechanic, who wanders forth on a holiday to breathe the fresh air of the country, finds no spot of green on which to rest his feet, or to see his children run about and gather flowers, and does but exchange the air of the manufactory for the dust of the road. Public sports, games, and amusements, there are none; and the close rooms of alehouses are the resort of the workmen in unemployed hours. The refreshment of a bath is unknown to them: if they bathe near a town, they are of course liable to punishment; and public baths are not to be found, or, if existing, are only for the richer class.* Mr. Buckingham's bill authorises the mayor of any town, on the requisition of fifty rate-payers, to call a meeting for the purpose of considering the expediency of establishing walks, baths, &c.; and we sincerely hope that these objects, and all that concerns the public health, will obtain due consideration from the new town-councils. If two thirds of the rate-payers present at such meeting are in favour of the proposal, small assessments may be levied to carry it into effect, under the direction of a committee, assisted by the donations of the wealthier classes.

It is proposed that the public walks be planted with trees and shrubs, and ornamented with fountains or running water. Play-grounds for gymnastic exercises, as cricket, archery, &c., are also to be established, under certain regulations, which will preclude brutal and degrading sports. Baths of every description are to be provided, including schools in which swimming will be taught. No beer, wine, spirits, or intoxicating drinks of any kind, are to be admitted. As, in the neighbourhood of some towns, there are already open spaces which would be suitable for such purposes, the committee are to have the power of granting loans for the improvement of such walks, &c. as are already in existence.

Of the excellence of this design we believe there can be but one opinion. If heartily entered into, it might conduce greatly to the health, cheerfulness, and contentment of large classes of people, who now know no recreation, or none that is really serviceable to them. The question is, however, how far the people of this country are prepared to enjoy such advantages, even if offered to them; and whether they are yet refined enough to take diversion in public walks without a propensity to mischief. It is feared that the grown-up men would quarrel, that the boys would destroy the fountains, and that decent women would be reluctant to enter the walks at all. With all these objections, however, which may not be so real as is supposed, we shall be glad to see Mr. Buckingham's bill carried into effect. Combined with another bill, introduced by the same gentleman, for the promotion of public scientific and literary institutions, libraries, museums, &c., it may tend to improve and raise a class of our population long too much neglected. In this respect, also, England is singularly behind. There are few country towns on the continent so barren of all the means of improvement as many or most of the county towns of our country. Upon the causes of this apparent general indifference to the means of acquiring literary and scientific information, it is not our business to enter: we trust, however, that it will soon give place to a different feeling. In the mean time, only good can arise from the establishment of public baths and public walks, and from a careful attention to all the circumstances in different localities which can affect the health and comfort of the people at large.

* We understand that public baths are about to be established at Sheffield, for the accommodation of the working classes.

